# Unraveling the Serum Metabolomic Profile of Post-partum Depression

**DOI:** 10.3389/fnins.2019.00833

**Published:** 2019-08-23

**Authors:** Zoe Papadopoulou, Angeliki-Maria Vlaikou, Daniela Theodoridou, Chrysoula Komini, Georgia Chalkiadaki, Marina Vafeiadi, Katerina Margetaki, Theoni Trangas, Chris W. Turck, Maria Syrrou, Leda Chatzi, Michaela D. Filiou

**Affiliations:** ^1^Laboratory of Biology, Faculty of Medicine, School of Health Sciences, University of Ioannina, Ioannina, Greece; ^2^Laboratory of Biochemistry, Department of Biological Applications and Technology, School of Health Sciences, University of Ioannina, Ioannina, Greece; ^3^Department of Social Medicine, Faculty of Medicine, University of Crete, Heraklion, Greece; ^4^Proteomics and Biomarkers, Department of Translational Research in Psychiatry, Max Planck Institute of Psychiatry, Munich, Germany; ^5^Department of Preventive Medicine, University of Southern California, Los Angeles, CA, United States; ^6^Department of Stress Neurobiology and Neurogenetics, Max Planck Institute of Psychiatry, Munich, Germany

**Keywords:** PPD, early life stress, pregnancy, psychiatric disorders, metabolomics, biomarkers, serum

## Abstract

Post-partum depression (PPD) is a severe psychiatric disorder affecting ∼15% of young mothers. Early life stressful conditions in periconceptual, fetal and early infant periods or exposure to maternal psychiatric disorders, have been linked to adverse childhood outcomes interfering with physiological, cognitive and emotional development. The molecular mechanisms of PPD are not yet fully understood. Unraveling the molecular underpinnings of PPD will allow timely detection and establishment of effective therapeutic approaches. To investigate the underlying molecular correlates of PPD in peripheral material, we compared the serum metabolomes of an in detail characterized group of mothers suffering from PPD and a control group of mothers, all from Heraklion, Crete in Greece. Serum samples were analyzed by a mass spectrometry platform for targeted metabolomics, based on selected reaction monitoring (SRM), which measures the levels of up to 300 metabolites. In the PPD group, we observed increased levels of glutathione-disulfide, adenylosuccinate, and ATP, which associate with oxidative stress, nucleotide biosynthesis and energy production pathways. We also followed up the metabolomic findings in a validation cohort of PPD mothers and controls. To the very best of our knowledge, this is the first metabolomic serum analysis in PPD. Our data show that molecular changes related to PPD are detectable in peripheral material, thus paving the way for additional studies in order to shed light on the molecular correlates of PPD.

## Introduction

Depression, the leading cause of disability worldwide (WHO, 2015), is more prevalent in women than men (Van de Velde et al., 2010; Rich et al., 2013). PPD is the most common psychiatric disorder in women after childbirth, with an increasing risk occurring during the first post-partum year (Gaillard et al., 2014). Although most women suffering from PPD show mild symptoms for a short period of time that gradually disappear, a percentage of women experiences heavier depressive symptoms (even post-partum psychosis) and need pharmacological treatment and psychotherapy (Leigh and Milgrom, 2008). Lack of psychiatric/therapeutic care may have dramatic consequences for the mother and the whole family (Pollock and Percy, 1999; Evins et al., 2000; Kurki et al., 2000). PPD has been also demonstrated to affect the development of the newborn with lasting effects to adulthood (Reck et al., 2004). Psychological and socio-economic factors have been implicated in the onset of PPD during the postnatal period. Socio-demographic variables, such as social support and high maternal educational level, have been negatively correlated with PPD, whereas young maternal age and smoking positively correlated with PPD (deCastro et al., 2011; Katon et al., 2014). Moreover, history of depression, anxiety and adverse life events are PPD risk factors (Verreault et al., 2014).

At the molecular level, hormonal changes have been described in relation to PPD. At birth, progesterone and estrogen levels significantly increase compared to pregnancy (Kumar and Magon, 2012; O’Hara and Wisner, 2014). Progesterone and estrogen levels are reduced post-partum and their variation has been implicated in mood changes (Hendrick et al., 1998). Active forms of estrogens (estradiol and estriol) are produced by the placenta and increase during pregnancy. Cortisol, a glucocorticoid steroid hormone, is an important marker of the stress response and is implicated in depression and PPD (Seth et al., 2016). A correlation between cortisol and PPD is well-established (Ehlert et al., 1990; Okano and Nomura, 1992; Taylor et al., 1994). Women with depressive symptoms after birth show elevated cortisol levels compared to controls (Okano and Nomura, 1992), although other studies have reported a negative correlation between cortisol levels and PPD, 4–6 weeks and 12 months post-partum (Parry et al., 2003; Groer and Morgan, 2007). In addition, the association between PPD and high levels of cortisol in hair during pregnancy has been proposed as a predictive marker for PPD (Diego et al., 2004). Amino acid metabolism has a key role in pregnancy and in the development of PPD (Duan et al., 2018). Lower plasma tryptophan levels are reported in PPD (Ogawa et al., 2014) and a poor tryptophan diet may induce depressive symptoms (Ellenbogen et al., 1999). Polymorphisms in genes of the tryptophan-serotonin pathway could affect the sensitivity to stress during pregnancy and post-partum, having an impact on the development of depressive symptoms (Figueiredo et al., 2015; Duan et al., 2018). Furthermore, altered levels of neurosteroids and GABA in PPD women suggest that their interaction could play a key role in the development of depression during pregnancy and post-partum (Deligiannidis et al., 2016).

Besides these molecular changes, the implicated molecular mechanisms at a systemic level in PPD are incompletely understood. To identify molecular signatures of PPD in peripheral material, we performed a detailed metabolomic analysis in serum of women diagnosed with PPD compared to controls recruited from the same geographical region. This is one of the very few metabolomic studies available for PPD.

## Materials and Methods

### Human Cohorts

This is a study within the Rhea pregnancy child cohort in Crete, Greece (Chatzi et al., 2017). The study and validation populations were Caucasian pregnant women who live in Heraklion, Crete. Women were contacted at the first and third trimester of pregnancy, at birth, and at 8 weeks post-partum. Face-to-face structured interviews, together with self-administered questionnaires and medical records, were used to obtain information on several psychosocial, dietary and environmental exposures during pregnancy and post-partum. The metabolomic study cohort included women diagnosed with PPD (EPDS score ≥ 13, *n* = 10) and women with no PPD (EPDS score < 13, *n* = 10) (Supplementary Table S1). For further investigation of the metabolomic analysis results a validation cohort was studied, including eight women diagnosed with PPD (EPDS score ≥ 13) and seven women with no PPD (EPDS score < 13) (Supplementary Table S2). All women were 20–35 years old, non-smokers, non-obese (BMI < 35) and became pregnant between February 2007 and February 2008. Twin pregnancies, women under fertilization treatment, women with gestational diabetes, preeclampsia and women with psychological disorders before or during pregnancy were excluded from the study. The study was approved by the Ethical Committee of the University Hospital in Heraklion, Crete, Greece. Written informed consent was obtained from all participants.

### Protocol for Assessing Depression

Maternal depressive symptoms were assessed (antenatally at 28–32 weeks of gestation and postnatally at 8 weeks post-partum) using the EPDS as previously described (Cox et al., 1987). The EPDS is a widely used 10-item, self-reported questionnaire providing an indication of the severity of mother’s mood during the past 7 days. Items are rated on a 4-point Likert scale ranging from 0 (not at all) to 3 (most of the time) and refers to depressed mood, anhedonia, guilt, anxiety and suicidal ideation (possible range 0–30). A cut-off score of ≥ 13 on the EPDS has been found to identify probable clinical postnatal depression with a sensitivity of 86% and a specificity of 78% (Matthey et al., 2006). This cut-off is also consistent with previous work in our cohort (Vivilaki et al., 2009; Chatzi et al., 2011; Koutra et al., 2014, 2017, 2018). The EPDS has been translated and validated for the Greek population by two research groups (Leonardou et al., 2009; Vivilaki et al., 2009) and showed a very high overall internal consistency.

### Serum Sample Collection

Blood samples were collected from pregnant women in Vacutainer SST Plastic Serum Tubes (BD 367958). Median gestational age at blood collection was the 14th week. To isolate serum, samples were centrifuged immediately after blood sampling collection for 10 min at 2500 rpm, room temperature. Serum samples were stored in 0.5 ml aliquots into cryovial sterile tubes at −80°C.

### Serum Sample Preparation

Serum metabolites were extracted with a fourfold excess (v/v) of 100% cold methanol as previously described (Filiou et al., 2014). After vortexing for 2 min, samples were incubated on dry ice for 2 h and centrifuged (2053 *g*, 100 min, 4°C). Supernatants were filtered using 0.22 μm SpinX ultrafiltration tubes (Corning, NY, United States), the filtrates were lyophilized and stored at −80°C for metabolomic analysis.

### Mass Spectrometry-Based Metabolomics

Serum metabolite extracts (100 μl per subject) were analyzed at the Metabolomics Core, Beth Israel Deaconess Medical Center (Harvard Medical School) by a SRM-based targeted metabolomics platform using a 5500 QTRAP triple quadrupole mass spectrometer coupled to a Prominence UFCL HPLC system, as previously described (Filiou et al., 2014). This platform quantifies the levels of up to 300 metabolites involved in major metabolic pathways (Yuan et al., 2012).

### Western Blot

For assessing the levels of the antioxidant enzyme Prdx3 in the validation cohort, Western blot analysis was performed as previously described (Filiou et al., 2010) with slight modifications. Briefly, protein content was measured by Bradford Assay. From each serum sample, 25 μg were diluted in RIPA buffer (1:2, v/v), electrophorized and electrotransferred with a semi-dry, *trans-*blot turbo transfer system (Bio-Rad, Hercules, CA, United States). Membranes were incubated with an anti-Prdx3 primary antibody (Abcam ab16751, mouse monoclonal, 1:2000) and an anti-mouse secondary antibody (sc-Santa Cruz Biotechnology, Heidelberg, Germany). Signal intensity was measured using ImageJ. Equal total protein loading was ensured by Coomassie gel staining and signal intensity comparison.

### Total Antioxidant Capacity

The determination of total antioxidant capacity (TAC) in serum samples of the validation cohort, was performed as previously described (Ciuti and Liguri, 2017). Briefly, serum samples and glutathione (used as calibrator) were mixed 1:5 (v/v) with a pre-heated chromogenic reagent as described (Ciuti and Liguri, 2017) in a multi-well plate. Absorbance at 630 nm was determined in a UT2100C microplate reader (MRC, Holon, Israel) and measurements were taken at 20 and 120 s after the last reagent dispensing. TAC quantification was performed as previously described (Ciuti and Liguri, 2017).

### Statistical Analysis

#### Metabolomic Study and Validation Cohort Demographic Data Analysis

A descriptive analysis of the study population characteristics was conducted. Categorical variables are presented as *N*(%) while continuous variables are presented as mean ± SD. We then compared the characteristics between PPD and control women and between the metabolomic study and validation groups utilizing Fisher’s exact test and Student’s *t*-test (*p* < 0.05).

#### Metabolomic Data Analysis

Metabolomic data analysis was performed by Metaboanalyst^1^ (v4.0) (Xia et al., 2009). Using the mass spectrometry-based metabolomics platform, 302 features were quantified. For metabolites measured both in positive and negative ion mode, only the measurement with the higher intensities across samples was included. In total, 285 metabolites were considered for analysis. Of those, metabolites with > 50% missing values were excluded. For the remaining metabolites, missing values were replaced by default small values in Metaboanalyst. Metabolite data were median-normalized, log-transformed and pareto-scaled. To evaluate the discriminative features between the PPD and control group we used the supervised PLS-DA feature in Metaboanalyst. To identify individual metabolite level changes between the PPD and control groups, we employed the SAM in Metaboanalyst using the siggenes R package. FDR was used to correct for multiple comparisons and the cut-off for the adjusted *p*-value (*q*-value) was set at 0.1.

#### Multivariable Analysis

For the quantified metabolites (normalized values) with altered levels in PPD compared to controls in the metabolomic study cohort, we performed linear regression analysis to further evaluate the observed associations adjusting for potential confounding factors. Using standard bivariate statistical tests (Fisher’s exact test, Student’s *t*-test, Pearson correlation coefficient) we identified variables associated with either the selected metabolites or with PPD at 10% level and we included those variables in the linear models. The selected covariates were age at blood sampling, working during pregnancy, educational status and pre pregnancy overweight (Model 1). In a second model, we included clinical characteristics, namely history of dyslipidemia and history thyroid disease (Model 2). Effect estimates are presented in terms of beta coefficients and 95% confidence intervals (95% CIs). Multivariable analyses were performed in Stata 13^2^.

#### Western Blot and TAC Data Analysis

Western blot and TAC statistical analysis was performed by GraphPad Prism7 (GraphPad, San Diego, CA, United States) using the Mann–Whitney non-parametric statistical test (*p* < 0.05). Data are presented as mean ± SEM.

## Results

### Metabolomic Study and Validation Cohorts

Detailed information on the demographic characteristics of the metabolomic study and validation cohorts is provided in Supplementary Tables S1, S2, respectively. Briefly, regarding the metabolomic study cohort, the mean (SD) age of the participating women was 29.1 (3.8) years and the mean (SD) BMI was 23.2 (3.5) kg/m^2^ (Supplementary Table S1). Validation cohort characteristics were similar (Supplementary Table S2). To ensure that the metabolomic study and validation cohorts were not demographically different, a comparison between the two cohorts was performed showing no differences in demographic characteristics (all *p*-values > 0.05, Supplementary Table S3).

### Altered Levels of Serum Metabolites in PPD

**FIGURE 1:**
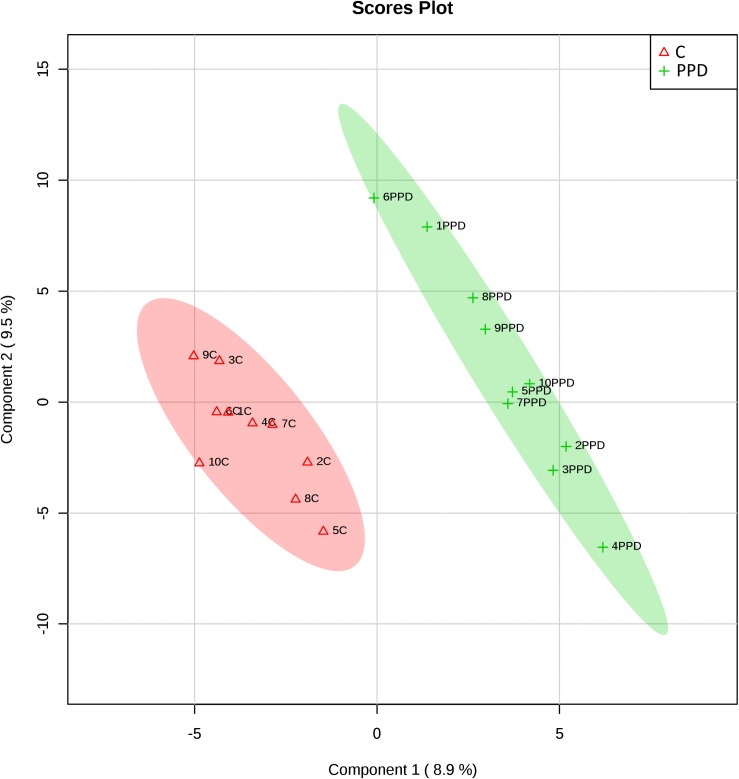
Score plots of metabolomic profiles of women suffering from PPD compared to controls in the metabolomic study cohort. C: control.

**FIGURE 2:**
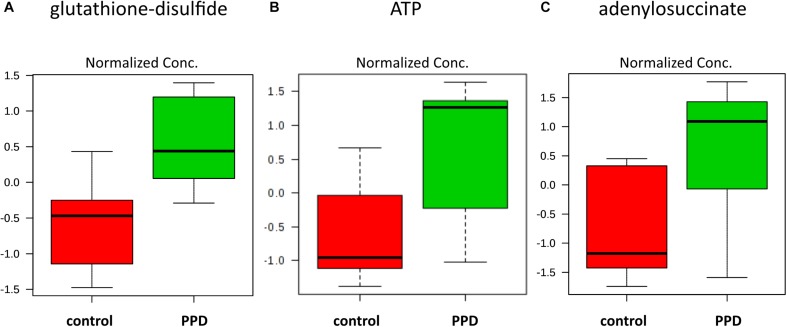
Increased metabolite levels in PPD serum compared to controls in the metabolomic study cohort **(A)** glutathione-disulfide (*d* = 2.6504, *SD* = 0.2543, and *q* = 0.0567) **(B)** ATP (*d* = 2.3833, *SD* = 0.3788, and *q* = 0.0567) **(C)** adenylosuccinate (*d* = 2.2390, *SD* = 0.4445, and *q* = 0.0825).

**FIGURE 3:**
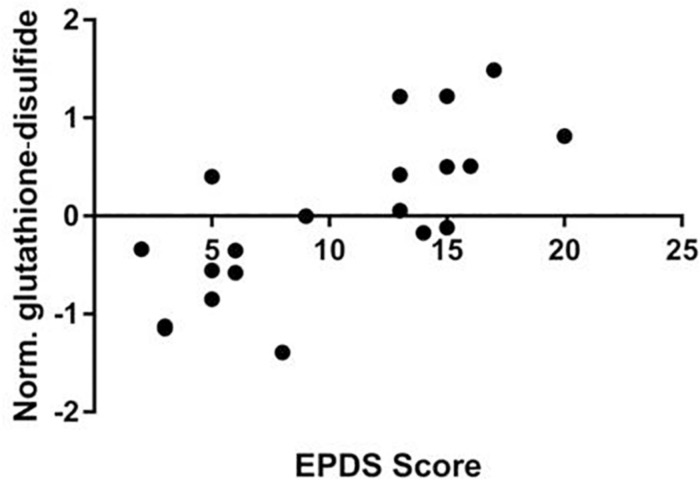
Correlation of EPDS scores and normalized glutathione-disulfide levels in the metabolomic study cohort (Pearson *r* = 0.7449, *p* < 0.001).

We performed a targeted metabolomic analysis in serum of pregnant women who developed PPD compared to unaffected women from the same geographical region. Quantification raw data for all 285 metabolites considered for the analysis are shown in Supplementary Table S4. The PLS-DA separation score plots of the two groups are shown in Figure 1. We identified three serum metabolites with altered levels in women with PPD compared to the control group. These included glutathione-disulfide, adenylosuccinate, and ATP. All three were found in elevated levels in PPD compared to the control group (Figure 2). These metabolites are associated with oxidative stress, nucleotide biosynthesis and energy production, respectively. We also found a positive association between normalized glutathione-disulfide levels with the continuous EPDS score (Pearson *r* = 0.7449, *p* < 0.001) (Figure 3). The results of the multivariable linear regression analysis are presented in Table 1. After adjustment for demographic characteristics, the observed associations remained significant. PPD was associated with higher levels of adenylosuccinate, glutathione-disulfide and ATP. The results remained similar after the adjustment for clinical characteristics with the exception of adenylosuccinate, where significance was lost.

**TABLE 1:** Adjusted associations between selected metabolites and PPD.

	**Model 1**		**Model 2**	
		
	**Beta (95% CI)**	***p*-Value**	**Beta (95% CI)**	***p*-Value**
ATP	1.12 (0.44, 1.80)	0.004	1.78 (0.99, 2.56)	<0.001
Glutathione-disulfide	0.94 (0.31, 1.57)	0.006	0.94 (0.00, 1.88)	0.051
Adenylosuccinate	1.34 (0.01, 2.68)	0.049	0.59 (−1.27, 2.44)	0.501

*Model 1: Adjusted for age at blood sampling, working and educational status and overweight pre pregnancy. Model 2: Model 1, additionally adjusted for history of dyslipidemia and history of thyroid disease.*

### Investigation of Oxidative Stress-Related Changes in a Validation Cohort

**FIGURE 4:**
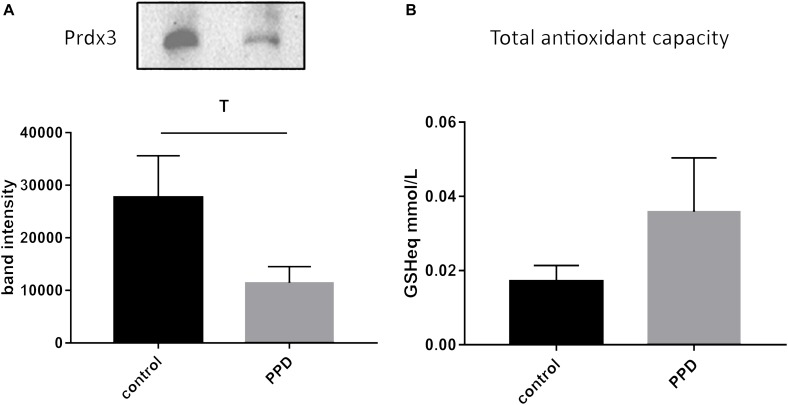
**(A)** A trend toward decreased expression of Prdx3 (*p* = 0.0939) in PPD (11437 ± 3068) compared to controls (27685 ± 7926) serum in the validation cohort. **(B)** No changes in total antioxidant capacity (expressed in terms of mmol/L of reduced glutathione, GSHeq, *p* = 0.554) in the validation cohort between PPD (0.0359 ± 0.0145) and controls (0.0171 ± 0.0042).

Given the positive correlation of glutathione-disulfide with the continuous EPDS score, we then went on to further investigate oxidative stress-related changes in serum of a validation cohort of women diagnosed with PPD vs. controls from the same geographical area (Supplementary Table S2). We assessed the levels of Prdx3, a member of the peroxiredoxin family of antioxidant enzymes, and found a trend for decreased Prdx3 expression in PPD samples (Figure 4A and Supplementary Figure S1). In the validation cohort, we also assessed the serum TAC and found no difference between the PPD and control samples (Figure 4B).

## Discussion

The aim of this work was to identify molecular signatures for PPD by comparing the serum metabolomic profiles of women with PPD vs. control subjects. Multi-omics approaches have the potential to shed light on molecular mechanisms of psychiatric disorders in a high-throughput and data-driven manner (Filiou, 2015). Metabolomic analyses provide valuable information for dissecting neuropsychiatric conditions, as the metabolome correlates directly with the phenotype and is very sensitive to environmental stressors and changes (Quinones and Kaddurah-Daouk, 2009; Turck and Filiou, 2015). Metabolomic approaches have been used to study depression both in patient cohorts and animal models (Pan et al., 2018; Zhang et al., 2018). To the best of our knowledge, this is the first metabolomics study in serum of PPD.

We report altered levels of three serum metabolites: glutathione-disulfide, adenylosuccinate, and ATP. Glutathione is a low molecular mass thiol and one of the most important endogenous antioxidant compounds (Freed et al., 2017). Under oxidative conditions, glutathione (reduced form) is converted into glutathione-disulfide (oxidized form). The glutathione/glutathione-disulfide ratio is considered as a prognostic factor for oxidative stress (Tietze, 1969; Freed et al., 2017). Both glutathione levels and the glutathione-disulfide/glutathione ratio of premature infants with idiopathic respiratory distress syndrome are increased compared to control newborns (Giustarini et al., 2016). Maternal prenatal distress was also shown to reduce placental glutathione/glutathione-disulfide ratios (Chang et al., 2016). Oxidative stress and pertinent markers are increased in patients with major depressive disorder (Dowlati et al., 2010; Black et al., 2015; Lindqvist et al., 2017). The correlation between oxidative stress and depression is not yet fully understood, however, the brain is reported to be particularly sensitive to oxidative damage due to elevated oxygen levels and free radicals (Ng et al., 2008). Free radicals may affect methylation patterns by hydroxylation of pyrimidines and 5-methylcytosine (Lewandowska and Bartoszek, 2011) and influence histone modifications through intracellular metabolites such as acetyl-CoA, ketoglutarate, NAD^+^, and S-adenosylmethionine (Simpson et al., 2012). Adenylosuccinate is an intermediate in nucleotide biosynthesis, involved in the conversion of inosine monophosphate (IMP) to adenosine monophosphate (AMP) (Gooding et al., 2015). ATP is the main cellular energy currency. Astrocyte-derived ATP was shown to modulate depression-like behaviors and brain ATP levels were lower in mice susceptible to chronic social defeat (Cao et al., 2013).

To follow up on the glutathione-disulfide level changes that correlated with the depressive status of the patients, we further investigated oxidative stress-related alterations in a validation cohort of PPD patients and controls. We found a trend for decreased expression of the antioxidant enzyme Prdx3 and an overall unchanged total antioxidant status of the PPD patients compared to controls in the validation cohort. Decreased expression of Prdx3 in PPD is in line with decreased expression of glutathione, which may result from the increased levels of glutathione-disulfide. It should be, however, noted that the complex interplay of various antioxidants performing complementary antioxidant functions may result in an overall unchanged TAC. From a technical perspective, immunochemical and colorimetric biochemical methods show significantly lower sensitivity compared to mass spectrometry-based metabolomics. The ability to identify changes by metabolomics which are undetectable by conventional biochemical methodologies highlights the need to use metabolomic approaches for the identification of low fold metabolite level changes. This is of particular interest for multifactorial disorders such as PPD, which are characterized by mild changes in multiple factors. In addition, the high sensitivity of mass spectrometry-based metabolomics is more appropriate for investigating peripheral material in brain disorders, where we do not expect to see dramatic fold changes, as brain-related pathophysiology alterations are attenuated in the periphery.

Metabolomic studies are indeed scarce in the context of PPD. A recent work compared urinary metabolites of PPD women, post-partum women with no PPD and healthy controls by gas-chromatography mass spectrometry-based metabolomics. In this study, 68 metabolites were identified and a panel of five metabolites was identified (formate, succinate, 1-methylhistidine, α-glucose, and dimethylamine), which was able to discriminate the PPD group. Interestingly, succinate is a precursor of adenylosuccinate, which was also found elevated in PPD in our study (Lin et al., 2017). A second study investigating the urine metabolome of PPD subjects compared to controls using untargeted mass spectrometry-based metabolomics indicated altered metabolomic profiles between the two groups (Zhang et al., 2019). A targeted steroid metabolome approach based on gas chromatography-mass spectrometry in maternal blood samples from PPD women revealed that an interplay of maternal derived testosterone and fetus derived estrogens may affect mood changes (Parizek et al., 2014).

Despite the small size of the population analyzed in this study, this is a well-characterized cohort with homogeneous demographic characteristics (women living in the same region and becoming pregnant at the same time). Future studies in larger sample sizes and other PPD cohorts are required to validate our results. The investigation of additional enzymes involved in antioxidant defense and biosynthetic processes will shed light on the role of these pathways in PPD pathogenesis. Combining metabolomic with genetic and epigenetic data might reveal risk markers of early stressful experiences. The acquired knowledge will be valuable for estimating the risk for future health disorders of the “stressed” newborns based on their individual genetic, epigenetic and metabolomic profiles.

Importantly, our data show that PPD-induced molecular changes are detectable in peripheral material. Identification of disease-related molecular correlates in peripheral material has been facing a series of challenges (Filiou and Turck, 2011), yet studies in the periphery are crucial for developing safe, non-invasive diagnostic and screening approaches for psychiatric disorders (Pinto et al., 2017). Intriguingly, a recent study in serum of post-partum women which had been exposed to different levels of childhood maltreatment, revealed a set of metabolites that could differ between varying levels of childhood trauma (Koenig et al., 2018). Our work may open up new perspectives for additional studies aiming at early detection and more accurate diagnosis using peripheral material for PPD and pave the way toward candidate biomarker identification.

## Data Availability

All datasets generated for this study are included in the manuscript and/or the Supplementary Files.

## Ethics Statement

The study was approved by the Ethical Committee of the University Hospital in Heraklion, Crete, Greece. Written informed consent was obtained from all participants.

## Author Contributions

ZP, A-MV, and CK performed the experiments. GC, MV, KM, and LC provided the clinical samples. TT, CT, MS, and MF designed and supervised the study. LC supervised the clinical sample and questionnaire collection. A-MV, DT, CK, KM, and MF analyzed the data. ZP, MS, and MF wrote the manuscript with input from all coauthors.

## Conflict of Interest Statement

The authors declare that the research was conducted in the absence of any commercial or financial relationships that could be construed as a potential conflict of interest.

## Footnotes

1 http://www.metaboanalyst.ca

2 https://www.stata.com/support/updates/stata13.html

## Supplementary Material

FIGURE S1 Western blot analysis of Prdx3 in the validation cohort.

TABLE S1 Characteristics of the metabolomic study cohort subjects according to PPD status, Rhea mother-child cohort, Crete, Greece. For demographics with no numerical values, an index is provided below each question. NAV denotes no available answer from the corresponding subject. NAP denotes a not applicablequestion for the corresponding subject.

TABLE S2 Characteristics of the validation cohort subjects according to PPD status, Rhea mother-child cohort, Crete, Greece. For demographics with no numerical values, an index is provided below each question. NAV denotes no available answer from the corresponding subject. NAP denotes a not applicable question for the corresponding subject.

TABLE S3 Comparison of subject characteristics in the metabolomic study and validation cohorts.

TABLE S4 Raw quantification data for measured metabolites (signal intensities). NA denotes non-quantified metabolites for the corresponding subject.

## Abbreviations

EPDS: Edinburgh postnatal depression scale
FDR: false discovery rate
PLS-DA: partial least squares-discriminant analysis
PPD: post-partum depression
Prdx3: peroxiredoxin-3
SAM: significance analysis of microarray
SEM: standard error of the mean
SRM: selected reaction monitoring
TAC: total antioxidant capacity.
