# Supplementary figures and images for: Unraveling the Serum Metabolomic Profile of Post-partum Depression
Source: Front Neurosci. 2019 Aug 23;13:833. doi: 10.3389/fnins.2019.00833 (PMC6716353; doi:10.3389/fnins.2019.00833)

**Figure S1**

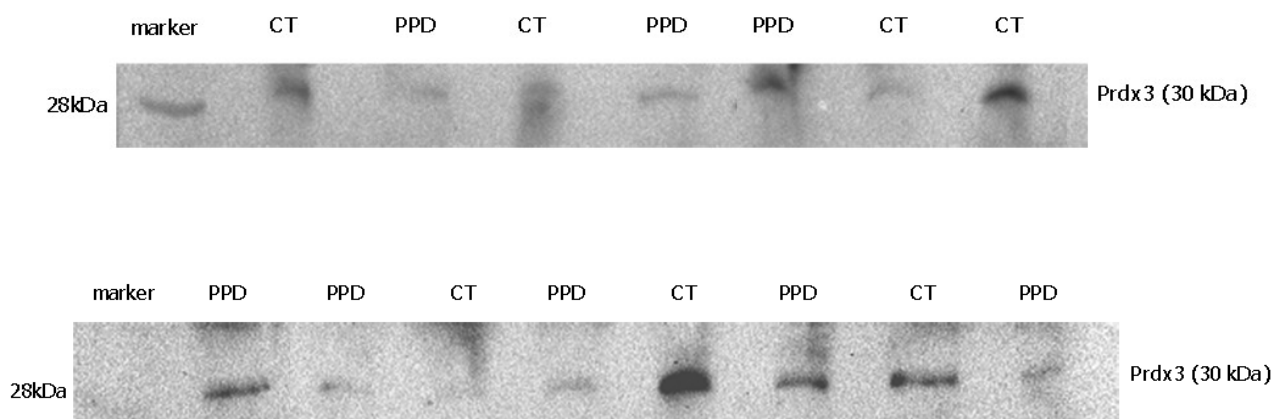

Supplement: FIGURE S1 — Western blot analysis of Prdx3 in the validation cohort. [file Image_1.pdf]
