# Supplementary material for: Unraveling the Serum Metabolomic Profile of Post-partum Depression
Source: Front Neurosci. 2019 Aug 23;13:833. doi: 10.3389/fnins.2019.00833 (PMC6716353; doi:10.3389/fnins.2019.00833)
Supplement: TABLE S1 — Characteristics of the metabolomic study cohort subjects according to PPD status, Rhea mother-child cohort, Crete, Greece. For demographics with no numerical values, an index is provided below each question. NAV denotes no available answer from the corresponding subject. NAP denotes a not applicablequestion for the corresponding subject. [file Table_1.docx]

**Table S1:** Characteristics of the metabolomic study cohort subjects according to post-partum depression (PPD) status, Rhea mother-child cohort, Crete, Greece

|  |  | Post-partum depression | |  |
| --- | --- | --- | --- | --- |
|  | Overall  N=20 | No  N=10 | Yes  N=10 |  |
|  | N (%) or  Mean ± SD | N (%) or  Mean ± SD | N (%) or  Mean ± SD | p-value* |
| Maternal age (years) | 29.1 ± 3.8 | 30.7 ± 3.5 | 27.6 ± 3.7 | 0.067 |
| BMI pre pregnancy (kg/m^2^) | 23.2 ± 3.5 | 22.8 ± 2.6 | 23.5 ± 4.3 | 0.695 |
| BMI at interview time (kg/m^2^) | 22.9 ± 3.4 | 22.7 ± 2.5 | 23.1 ± 4.3 | 0.798 |
| Marital status |  |  |  | 0.474 |
| Married | 16 (80.0) | 8 (80.0) | 8 (80.0) |  |
| Engaged | 2 (10.0) | 2 (20.0) | 0 (0.0) |  |
| Single | 1 (5.0) | 0 (0.0) | 1 (10.0) |  |
| NAV | 1 (5.0) | 0 (0.0) | 1 (10.0) |  |
| Working during pregnancy |  |  |  | 0.179 |
| No | 10 (50.0) | 3 (30.0) | 7 (70.0) |  |
| Yes | 10 (50.0) | 7 (70.0) | 3 (30.0) |  |
| Maternal education |  |  |  | 0.809 |
| Low | 2 (10.0) | 1 (10.0) | 1 (10.0) |  |
| Medium | 10 (50.0) | 4 (40.0) | 6 (60.0) |  |
| High | 8 (40.0) | 5 (50.0) | 3 (30.0) |  |
| Paternal education |  |  |  | 0.820 |
| Low | 4 (20.0) | 2 (20.0) | 2 (20.0) |  |
| Medium | 11 (55.0) | 5 (50.0) | 6 (60.0) |  |
| High | 4 (20.0) | 3 (30.0) | 1 (10.0) |  |
| NAV | 1 (5.0) | 0 (0.0) | 1 (10.0) |  |
| Planned pregnancy |  |  |  | 0.020 |
| Yes | 12 (60.0) | 9 (90.0) | 3 (30.0) |  |
| No | 8 (40.0) | 1 (10.0) | 7 (70.0) |  |
| Prenatal control |  |  |  | 0.474 |
| No | 2 (10.0) | 0 (0.0) | 2 (20.0) |  |
| Yes | 18 (90.0) | 10 (100.0) | 8 (80.0) |  |
| Hospitalization during pregnancy |  |  |  | 1.000 |
| No | 17 (85.0) | 8 (80.0) | 9 (90.0) |  |
| Yes | 1 (5.0) | 1 (10.0) | 0 (0.0) |  |
| NAV | 2 (10.0) | 1 (10.0) | 1 (10.0) |  |
| Previous pregnancy |  |  |  | 0.656 |
| No | 10 (50.0) | 4 (40.0) | 6 (60.0) |  |
| Yes | 10 (50.0) | 6 (60.0) | 4 (40.0) |  |
| PPD in previous pregnancy |  |  |  | 1.000 |
| No | 6 (30.0) | 3 (30.0) | 3 (30.0) |  |
| Yes | 1 (5.0) | 0 (0.0) | 1 (10.0) |  |
| NAV | 3 (15.0) | 3 (30.0) | 0 (0.0) |  |
| NAP | 10 (50.0) | 4 (40.0) | 6 (60.0) |  |
| History of dyslipidemia |  |  |  | 0.474 |
| No | 18 (90.0) | 10 (100.0) | 8 (80.0) |  |
| Yes | 2 (10.0) | 0 (0.0) | 2 (20.0) |  |
| History of thyroid disease |  |  |  | 0.211 |
| No | 17 (85.0) | 7 (70.0) | 10 (100.0) |  |
| Yes | 3 (15.0) | 3 (30.0) | 0 (0.0) |  |
| Exposure to second hand smoke during pregnancy |  |  |  | 0.370 |
| No | 11 (55.0) | 7 (70.0) | 4 (40.0) |  |
| Yes | 9 (45.0) | 3 (30.0) | 6 (60.0) |  |

*Chi-square (Fisher's exact test) for categorical variables; t-test for continuous variables
