# Supplementary material for: Unraveling the Serum Metabolomic Profile of Post-partum Depression
Source: Front Neurosci. 2019 Aug 23;13:833. doi: 10.3389/fnins.2019.00833 (PMC6716353; doi:10.3389/fnins.2019.00833)
Supplement: TABLE S2 — Characteristics of the validation cohort subjects according to PPD status, Rhea mother-child cohort, Crete, Greece. For demographics with no numerical values, an index is provided below each question. NAV denotes no available answer from the corresponding subject. NAP denotes a not applicable question for the corresponding subject. [file Table_2.DOCX]

**Table S2:** Characteristics of the validation cohort subjects according to post-partum depression (PPD) status, Rhea mother-child cohort, Crete, Greece

|  |  | Post-partum depression | |  |
| --- | --- | --- | --- | --- |
|  | Overall  N=15 | No  N=7 | Yes  N=8 |  |
|  | N (%) or  Mean ± SD | N (%) or  Mean ± SD | N (%) or  Mean ± SD | p-value* |
| Maternal age (years) | 26.6 ±4.5 | 24.9 ±4.8 | 28.1 ±4.0 | 0.172 |
| BMI pre pregnancy (kg/m^2^) | 24.1 ±3.9 | 22.7 ±4.1 | 25.4 ±3.5 | 0.204 |
| BMI at interview time (kg/m^2^) | 24.8 ±3.7 | 23.9 ±4.1 | 25.6 ±3.5 | 0.379 |
| Marital status |  |  |  | 1.000 |
| Married | 12 (80.0) | 6 (85.7) | 6 (75.0) |  |
| Engaged | 3 (20.0) | 1 (14.3) | 2 (25.0) |  |
| Working during pregnancy |  |  |  | 0.608 |
| No | 9 (60.0) | 5 (71.4) | 4 (50.0) |  |
| Yes | 6 (40.0) | 2 (28.6) | 4 (50.0) |  |
| Maternal education |  |  |  | 0.491 |
| Low | 4 (26.7) | 3 (42.9) | 1 (12.5) |  |
| Medium | 9 (60.0) | 3 (42.9) | 6 (75.0) |  |
| High | 2 (13.3) | 1 (14.3) | 1 (12.5) |  |
| Paternal education |  |  |  | 0.510 |
| Low | 6 (40.0) | 4 (57.1) | 2 (25.0) |  |
| Medium | 7 (46.7) | 2 (28.6) | 5 (62.5) |  |
| High | 2 (13.3) | 1 (14.3) | 1 (12.5) |  |
| Planned pregnancy |  |  |  | 0.103 |
| Yes | 7 (46.7) | 5 (71.4) | 2 (25.0) |  |
| No | 7 (46.7) | 1 (14.3) | 6 (75.0) |  |
| NAV | 1 (6.7) | 1 (14.3) | 0 (0.0) |  |
| Prenatal control |  |  |  | 1.000 |
| No | 5 (33.3) | 2 (28.6) | 3 (37.5) |  |
| Yes | 9 (60.0) | 4 (57.1) | 5 (62.5) |  |
| NAV | 1 (6.7) | 1 (14.3) | 0 (0.0) |  |
| Hospitalization during pregnancy |  |  |  | 1.000 |
| No | 12 (80.0) | 6 (85.7) | 6 (75.0) |  |
| Yes | 1 (6.7) | 0 (0.0) | 1 (12.5) |  |
| NAV | 2 (13.3) | 1 (14.3) | 1 (12.5) |  |
| Previous pregnancy |  |  |  | 0.619 |
| No | 8 (53.3) | 3 (42.9) | 5 (62.5) |  |
| Yes | 7 (46.7) | 4 (57.1) | 3 (37.5) |  |
| PPD in previous pregnancy |  |  |  | 0.619 |
| No | 7 (46.7) | 4 (57.1) | 3 (37.5) |  |
| NAP | 8 (53.3) | 3 (42.9) | 5 (62.5) |  |
| History of dyslipidemia |  |  |  | 0.467 |
| No | 14 (93.3) | 6 (85.7) | 8 (100.0) |  |
| Yes | 1 (6.7) | 1 (14.3) | 0 (0.0) |  |
| History of thyroid disease |  |  |  | 0.200 |
| No | 12 (80.0) | 7 (100.0) | 5 (62.5) |  |
| Yes | 3 (20.0) | 0 (0.0) | 3 (37.5) |  |
| Exposure to second hand smoke during pregnancy |  |  |  | 0.315 |
| No | 8 (53.3) | 5 (71.4) | 3 (37.5) |  |
| Yes | 7 (46.7) | 2 (28.6) | 5 (62.5) |  |

*Chi-square (Fisher's exact test) for categorical variables; t-test for continuous variables
