# Supplementary material for: Unraveling the Serum Metabolomic Profile of Post-partum Depression
Source: Front Neurosci. 2019 Aug 23;13:833. doi: 10.3389/fnins.2019.00833 (PMC6716353; doi:10.3389/fnins.2019.00833)
Supplement: TABLE S3 — Comparison of subject characteristics in the metabolomic study and validation cohorts. [file Table_3.DOCX]

**Table S3:** Comparison of subject characteristics in the metabolomic study and validation cohorts

|  | Metabolomic study cohort  N=20 | Validation cohort  N=15 |  |
| --- | --- | --- | --- |
|  | N (%) or  Mean ± SD | N (%) or  Mean ± SD | p-value* |
| Maternal age (years) | 29.1 (3.8) | 26.6 (4.5) | 0.080 |
| BMI pre pregnancy (kg/m^2^) | 23.2 (3.5) | 24.1 (3.9) | 0.446 |
| BMI at interview time (kg/m^2^) | 22.9 (3.4) | 24.8 (3.7) | 0.123 |
| Marital status |  |  | 0.798 |
| Married | 16 (80.0) | 12 (80.0) |  |
| Engaged | 2 (10.0) | 3 (20.0) |  |
| Single | 1 (5.0) | 0 (0.0) |  |
| NAV | 1 (5.0) | 0 (0.0) |  |
| Working during pregnancy |  |  | 0.734 |
| No | 10 (50.0) | 9 (60.0) |  |
| Yes | 10 (50.0) | 6 (40.0) |  |
| Maternal education |  |  | 0.187 |
| Low | 2 (10.0) | 4 (26.7) |  |
| Medium | 10 (50.0) | 9 (60.0) |  |
| High | 8 (40.0) | 2 (13.3) |  |
| Paternal education |  |  | 0.580 |
| Low | 4 (20.0) | 6 (40.0) |  |
| Medium | 11 (55.0) | 7 (46.7) |  |
| High | 4 (20.0) | 2 (13.3) |  |
| NAV | 1 (5.0) | 0 (0.0) |  |
| Planned pregnancy |  |  | 0.728 |
| Yes | 12 (60.0) | 7 (46.7) |  |
| No | 8 (40.0) | 7 (46.7) |  |
| NAV | 0 (0.0) | 1 (6.7) |  |
| Prenatal control |  |  | 0.097 |
| No | 2 (10.0) | 5 (33.3) |  |
| Yes | 18 (90.0) | 9 (60.0) |  |
| NAV | 0 (0.0) | 1 (6.7) |  |
| Hospitalization during pregnancy |  |  | 1.000 |
| No | 17 (85.0) | 12 (80.0) |  |
| Yes | 1 (5.0) | 1 (6.7) |  |
| NAV | 2 (10.0) | 2 (13.3) |  |
| Previous pregnancy |  |  | 1.000 |
| No | 10 (50.0) | 8 (53.3) |  |
| Yes | 10 (50.0) | 7 (46.7) |  |
| PPD in previous pregnancy |  |  | 0.853 |
| No | 6 (30.0) | 7 (46.7) |  |
| Yes | 1 (5.0) | 0 (0.0) |  |
| NAV | 3 (15.0) | 0 (0.0) |  |
| NAP | 10 (50.0) | 8 (53.3) |  |
| History of dyslipidemia |  |  | 1.000 |
| No | 18 (90.0) | 14 (93.3) |  |
| Yes | 2 (10.0) | 1 (6.7) |  |
| History of thyroid disease |  |  | 1.000 |
| No | 17 (85.0) | 12 (80.0) |  |
| Yes | 3 (15.0) | 3 (20.0) |  |
| Exposure to second hand smoke during pregnancy |  |  | 1.000 |
| No | 11 (55.0) | 8 (53.3) |  |
| Yes | 9 (45.0) | 7 (46.7) |  |

*Chi-square (Fisher's exact test) for categorical variables; t-test for continuous variables
